# Lessons learned for spine SABR?

**DOI:** 10.1016/j.ctro.2024.100728

**Published:** 2024-01-19

**Authors:** Stephane Thibodeau, Gregory Paulin, Fabio Ynoe Moraes

**Affiliations:** a Department of Oncology, Queen’s University Kingston Health Sciences Centre, Kingston, ON, Canada; b Department of Radiology and Oncology, University of São Paulo Medical School, São Paulo, Brazil

## Abstract

• SABR has shown survival benefits in oligometastatic cases, particularly in low-volume metastatic disease states.
• Spine SABR offers potential improvements in local control, and pain response for metastatic spine tumors.
• Technical requirements for SABR, like advanced image guidance and immobilization systems, are increasingly available.
• Current data suggests high local control rates (75–95%) and variable pain responses (40–90%) with SABR using single or multi-fraction regimens.
• Further randomized trials are needed to explore SABR applications in different scenarios and populations, while considering global health aspects for wider accessibility.

The putative benefits of stereotactic ablative radiotherapy (SABR) have taken the field of radiation oncology by storm. The horse may not truly have “left the barn” in what we have previously thought of as incurable disease in the case of selected, low-volume metastatic disease states (so called “oligometastatic”) in some histologies where clear survival benefits have been demonstrated in multiple randomized trials[1] with an aggressive primary- and metastasis-directed approach. The spine is a common site of (oligo)metastasis (∼30 %)[2], and the workhorse of palliative radiotherapy, the conventional parallel-opposed pair, is known to demonstrate modest improvements in pain control (∼60 %)[3] and frequent local recurrence (∼40–60 % at 1 year)[4]. The spine is thus seen as an ideal candidate for SABR-directed therapy, with the prospect of improving survival, local control, and pain response. Improving pain control rates could in turn lead to reduced needs for repeat treatments, which could improve access to health resources for a greater number of patients.

While technical requirements for the delivery of SABR require modern linacs with advanced image guidance solutions and robust immobilisation systems, these resources are becoming increasingly commonplace, particularly within North America, and while access continues to vary considerably by geography, this is expected to improve over the coming years. Although SABR usage has historically been found to be more prevalent among academic centers, there has been rapid uptake in SABR delivery by independent practitioners that further demonstrates just how accessible the technology has become within the broader community [5], [6].

Retrospective and non-randomized prospective data[7] have revealed 1- to 2-year local control rates on the order of 75–95 %, with variable pain responses ranging from 40 to 90 %. Doses in the literature have ranged from single to multi-fraction regimens. The CCTG SC.24 randomized phase II/III trial by Sahgal et. al.[8] revealed a positive primary endpoint of 3-month complete pain response, with more than a doubling of the response rate in patients treated with 24 Gy in 2 daily fractions compared to 20 Gy in 5 daily fractions (14 % to 35 %, p = 0.0002). However, with the recent publication of the NRG/RTOG 0631 phase III trial^9^ which revealed a negative primary endpoint, suggestive even of worse 3-month pain response comparing single fractions of 16–18 Gy versus 8 Gy, one would not be hard pressed to wonder whether enthusiasm for spine SABR is merely a matter of biased patient selection.

It is the perspective of these authors that such a conclusion is premature. There are key limitations of RTOG 0631 that bear examination. Firstly, there may be a difference in biological effectiveness between SRS and the fractionated technique employed in SC.24 (assuming a/b of 10, 24 Gy in 2 fractions has a BED of 52, and 16 Gy and 18 Gy single fractions have a BED of 41 and 50 respectively). Those familiar with the SABR literature, especially in the CNS context, will not be surprised that fractionation has been shown to result in superior outcomes, as increasing treatment volumes necessitates decreasing prescription doses to avoid overdosing sensitive organs at risk, though this is not without its own controversies[10].

Secondly, SC.24 used a consensus guideline-defined pain response with a validated pain assessment system (Brief Pain Inventory) whereas RTOG 0631 used the Numerical Rating Pain Scale, which while commonly used in practice is less well-validated in the palliative radiotherapy setting. RTOG 0631 also used a convoluted process of scoring using the patient’s report pain at an “index lesion” yet also accounted for progressive pain at a secondary, non-index site as non-response. This decreases the external validity of their pain assessment schema. However, it is worth noting that beyond 12 months, the SRS arm reported continued improvement in pain response, while the pain response appears to plateau in the conventional arm, though this difference is not statistically different.

Finally, RTOG 0631 does not report findings of local control. Local control is of paramount importance to the oligometastatic paradigm, and it remains unclear how SRS compares to conventional fractionated palliative radiotherapy in this regard. This limitation could in part be explained by differences in target volume delineation in RTOG 0631, with SABR delivered following Cox guidelines, and the conventional arm including one vertebrae above and below the target vertebrae. On the other hand, SC.24 used in both arms a marginal expansion of 1–2 cm each from CTV to PTV, and PTV to penumbra, with CTV being the entire target vertebrae. In addition, using a single fraction as was done in the conventional arm would not be considered a Canadian standard for patients with spine metastases with an expected survival greater than 3 months, as local control is likely improved with fractionation [11].

Taking the findings of RTOG 0631 at face value, it is worth considering the therapeutic ratio of potential local control benefit within the overall cure of a patient’s oligometastatic state, where small decreases in pain response potentially necessitating more analgesia could be a reasonable trade-off. It is of course worth emphasizing that such a trade-off would not be acceptable if the treatment intent were purely for palliation. We must as a discipline agree on the appropriate paradigm through which to view the role of spine SABR. Is the purpose of SABR really just about pain control, or does it possibly play a more important role in controlling disease as part of an oligometastatic paradigm of cure? The results of RTOG 0631 does not help us answer this question.

In the end, equipoise remains within the spine SABR literature, and further randomized trials are needed to elucidate its role compared to conventional radiotherapy. Going forward, we recommend that future spine SABR trials employ fractionated regimens rather than SRS and that they be powered not only for pain response/quality-of-life, but also for local control and survival in line with validating its role as part of an oligometastatic approach. In addition, trials should incorporate a global health lens so that the benefits of spine SABR are available to all, across low-, middle-, and high-income countries.

## Declaration of competing interest

The authors declare that they have no known competing financial interests or personal relationships that could have appeared to influence the work reported in this paper.
